# Supplementary material for: Microbial Communities in Gynecological Cancers and Their Association with Tumor Somatic Variation
Source: Cancers (Basel). 2023 Jun 23;15(13):3316. doi: 10.3390/cancers15133316 (PMC10340580; doi:10.3390/cancers15133316)
Supplement: Supplementary file 1 [file cancers-15-03316-s001.zip › metagenomics and variation - Supplemenatry 5-14-23.pdf]

cancers

MDPI

Article

Microbial Communities in Gynecological Cancers and Their Association with Tumor Somatic Variation

Jesus Gonzalez-Bosquet [1,2,*], Megan E. McDonald [1,2], David P. Bender [1,2], Brian J. Smith [3], Kimberly K. Leslie [4], Michael J. Goodheart [1,2] and Eric J. Devor [1,2]

1 Division of Gynecologic Oncology, Department of Obstetrics and Gynecology, University of Iowa Hospitals and Clinics, Iowa City, IA 52242, USA; megan-mcdonald@uiowa.edu (M.E.M.); david-bender@uiowa.edu (D.P.B.); michael-goodheart@uiowa.edu (M.J.G.); eric-devor@uiowa.edu (E.J.D.)
2 Holden Comprehensive Cancer Center, University of Iowa Hospitals and Clinics, Iowa City, IA 52242, USA
3 Department of Biostatistics, University of Iowa, Iowa City, IA 52242, USA; brian-j-smith@uiowa.edu
4 Division of Molecular Medicine, Department of Internal Medicine and Obstetrics and Gynecology, The University of New Mexico Comprehensive Cancer Center, Albuquerque, NM 87131, USA; kkleslie@salud.unm.edu
* Correspondence: jesus-gonzalezbosquet@uiowa.edu; Tel.: +1-319-356-2015

A.

	OR	padj
Thermococcus	0.02	2.7E-08
Acidaminobacter	0.02	1.8E-07
Bifidobacteriaceae	0.02	1.8E-07
unclassified_Desulfobacteraceae	0.03	2.4E-07
Geobacter	0.02	2.9E-07
Succinispira	0.04	2.9E-07
Dechloromonas	0.01	4.0E-07
Simplicispira	0.02	4.0E-07
Thermoanaerobacterium	0.03	4.0E-07
unclassified_Planctomycetaceae	0.02	4.0E-07
Flavobacterium	0.02	5.5E-07
Dysgonomonas	0.02	8.1E-07
Pediococcus	0.02	1.5E-06
unclassified_Chromatiaceae	0.04	1.7E-06
Thiobacillus	0.03	2.0E-06
Rhodopseudomonas	0.01	2.1E-06
Oxalobacter	0.05	4.0E-06
Planococcaceae_incertae_sedis	0.07	5.7E-06
Plesiomonas	0.02	5.7E-06
Serratia	0.01	6.2E-06
unclassified_Prolixibacteraceae	0.03	8.6E-06
Desulforhopalus	0.02	9.6E-06
Mucinivorans	0.01	1.2E-05
Methylotenera	0.02	1.3E-05
Actinomycineae	0.08	1.8E-05
unclassified_Beijerinckiaceae	0.04	1.8E-05
Massilia	0.04	1.9E-05
unclassified_Anaerolineaceae	0.01	2.3E-05
Cloacibacillus	0.03	4.1E-05
unclassified_Cytophagaceae	0.02	4.2E-05
Klebsiella	0.01	5.3E-05
Citrobacter	0.06	5.4E-05
Bacteriovorax	0.01	6.6E-05
Paenibacillus	0.08	7.4E-05
Paludibacter	0.01	1.1E-04

B.

	OR
Desulfobacter	7.0E-06
Desulfomicrobium	3.5E-22
Parabacteroides	8.2E-09
Proteus	1.2E-23

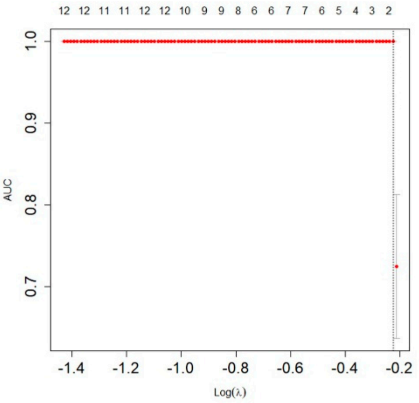

Supplementary Figure S1. Differential gene expression of 16S rRNA between EEC and normal endometrium.
A. Significant 16S rRNA expression between normal endometrium and ECC specimens (N=655) calculated by multiple univariate logistic regression analyses (N=112, p<0.05 FDR adjusted for multiple comparisons), using the *DESeq2* package. Showing the top 35 16S rRNA differentially expressed (out of 112). All significant 16S rRNA differentially expressed were associated with higher risk of ECC (OR >1). **B.** The lasso multivariate regression model included all 16S rRNA counts significant in the univariate analysis (N=112). In the upper panel: The lasso multivariate regression model: out of the 112 significant counts in the univariate analysis, four 16S sRNAs remained as informative for prediction ECC. All of them with reduced gene counts (OR <1) in EEC. In the lower panel: Graphic representation of the multivariate lasso analysis: superior margin reflects number of variables; left margin reflects performance of the model measured in AUC (area under the curve); lower margin reflects lambda tunning parameter chose by cross-validation to optimize the model. The optimized AUC was 1.0 (95% CI: 1.0, 1.0), between the dotted lines. Graphics were generated with R package *glmnet*.

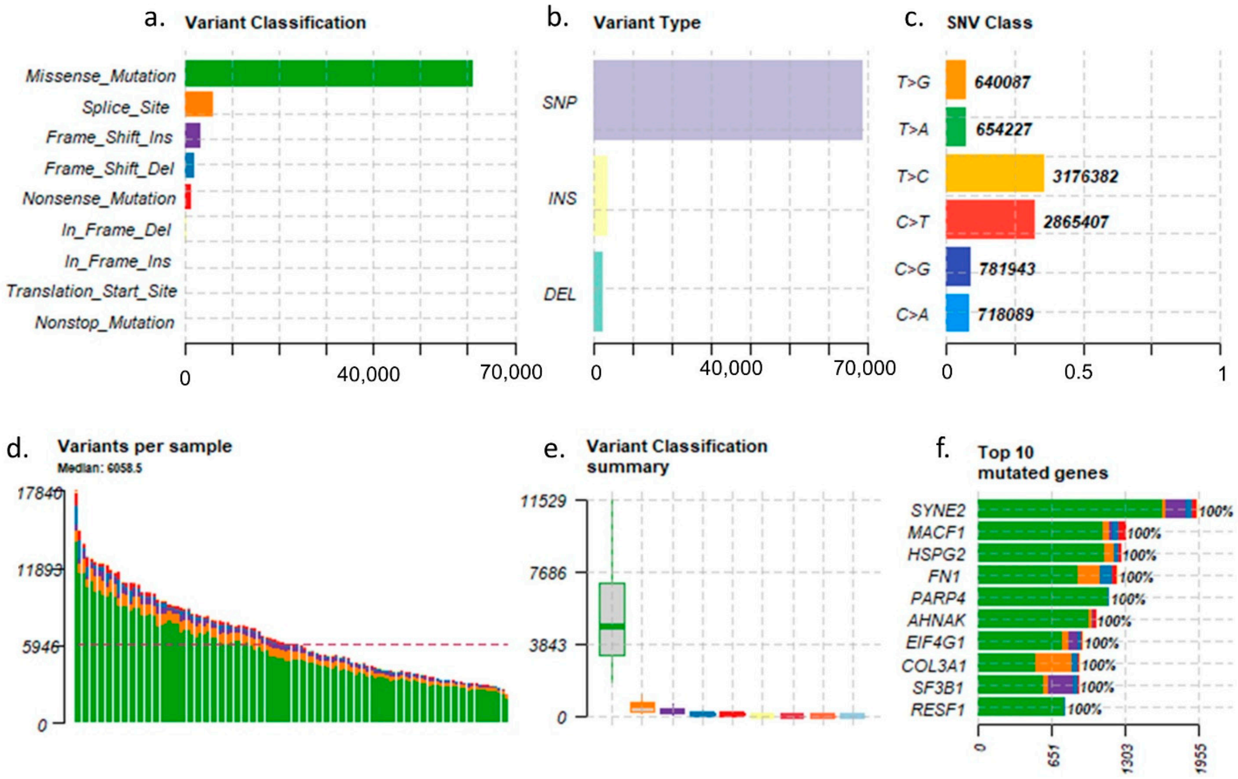

Supplementary Figure S2. Summary of Mutation Annotation Format (MAF) MAF files from HGSC samples. MAF files are used to store detected somatic variants. Representation of all single nucleotide variation (SNV) in the HGSC database:

a. SNV by classification: Frame-Shift deletion/insertion, In-Frame deletion/insertion, missense, non-sense or non-stop mutations, and splice site (x axis: total number);
b. SNV type: SNP, deletion or insertion (x axis: total number);
c. SNV class depending of nucleotide substitution (x axis: percentage);
d. SNVs per sample (y axis: total number);
e. Summary of SNV classification (based on a.), (y axis: total number);
f. Top 10 genes with number of SNVs (x axis: total number);

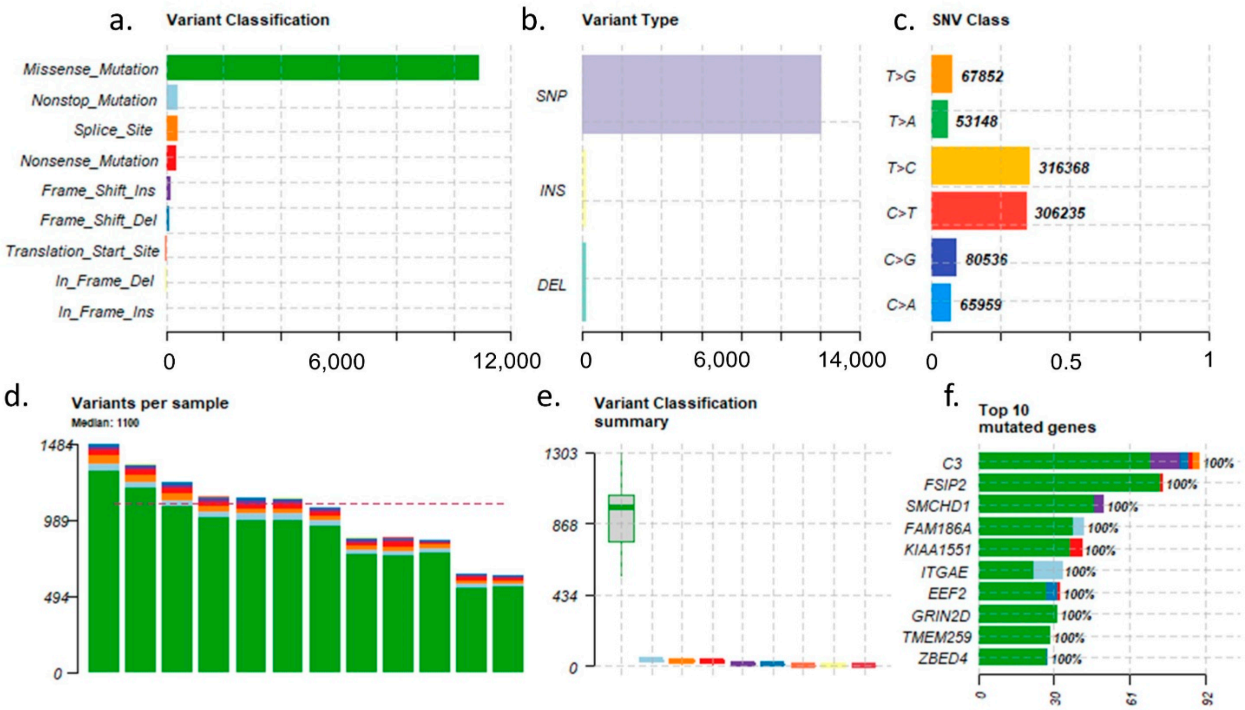

Supplementary Figure S3. Summary of Mutation Annotation Format (MAF) MAF files from normal fallopian tube samples. MAF files are used to store detected somatic variants. Representation of all single nucleotide variation (SNV) in the HGSC database:

g. SNV by classification: Frame-Shift deletion/insertion, In-Frame deletion/insertion, missense, non-sense or non-stop mutations, and splice site (x axis: total number);
h. SNV type: SNP, deletion or insertion (x axis: total number);
i. SNV class depending of nucleotide substitution (x axis: percentage);
j. SNVs per sample (y axis: total number);
k. Summary of SNV classification (based on a.), (y axis: total number);
l. Top 10 genes with number of SNVs (x axis: total number);

Supplementary Table S1. Difference in SNV counts between HGSC samples and normal tube samples. Table with 25 top genes (out of 593) with significant differences in SNV number between HGSC samples and tube samples (FDR adjusted p-value<0.05 to account for multiple comparisons).

Symbol	Cancer	Tube	adjusted p-value
CRISP2	0	12	8.26E-13
KIAA2012	4	11	6.17E-08
RGS7BP	5	11	1.30E-07
ANXA13	6	11	2.75E-07
RPL34	100	0	4.00E-07
C6	7	11	4.00E-07
PSAP	105	1	4.00E-07
CFAP299	4	10	6.24E-07
DNAH9	9	11	1.47E-06
CROCC2	5	10	1.47E-06
CFAP157	16	12	2.16E-06
DNAH12	10	11	2.16E-06
GBA3	10	11	2.16E-06
TUBA4B	10	11	2.16E-06
ANKDD1B	3	9	2.52E-06
DNAI2	3	9	2.52E-06
EFCAB6	17	12	2.52E-06
KIF19	1	8	2.72E-06
SCN1A	1	8	2.72E-06
CCDC187	11	11	3.00E-06
CEP126	18	12	3.40E-06
CCDC198	7	10	4.01E-06
CDHR3	7	10	4.01E-06
AC013470.2	4	9	5.30E-06
C16orf71	4	9	5.30E-06

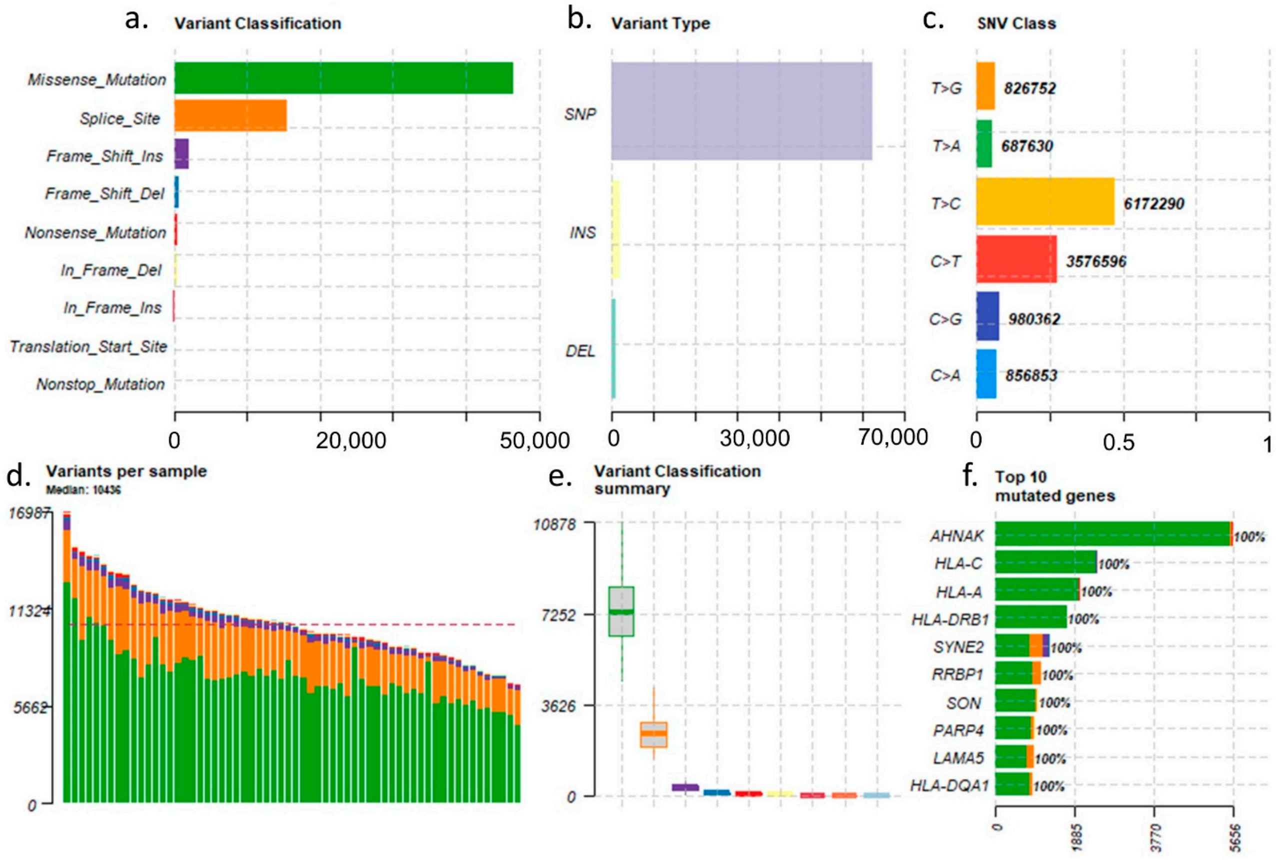

Supplementary Figure S4. Summary of Mutation Annotation Format (MAF) MAF files from EEC samples. MAF files are used to store detected somatic variants. Representation of all single nucleotide variation (SNV) in the HGSC database:

m. SNV by classification: Frame-Shift deletion/insertion, In-Frame deletion/insertion, missense, non-sense or non-stop mutations, and splice site (x axis: total number);
n. SNV type: SNP, deletion or insertion (x axis: total number);
o. SNV class depending of nucleotide substitution (x axis: percentage);
p. SNVs per sample (y axis: total number);
q. Summary of SNV classification (based on a.), (y axis: total number);
r. Top 10 genes with number of SNVs (x axis: total number);

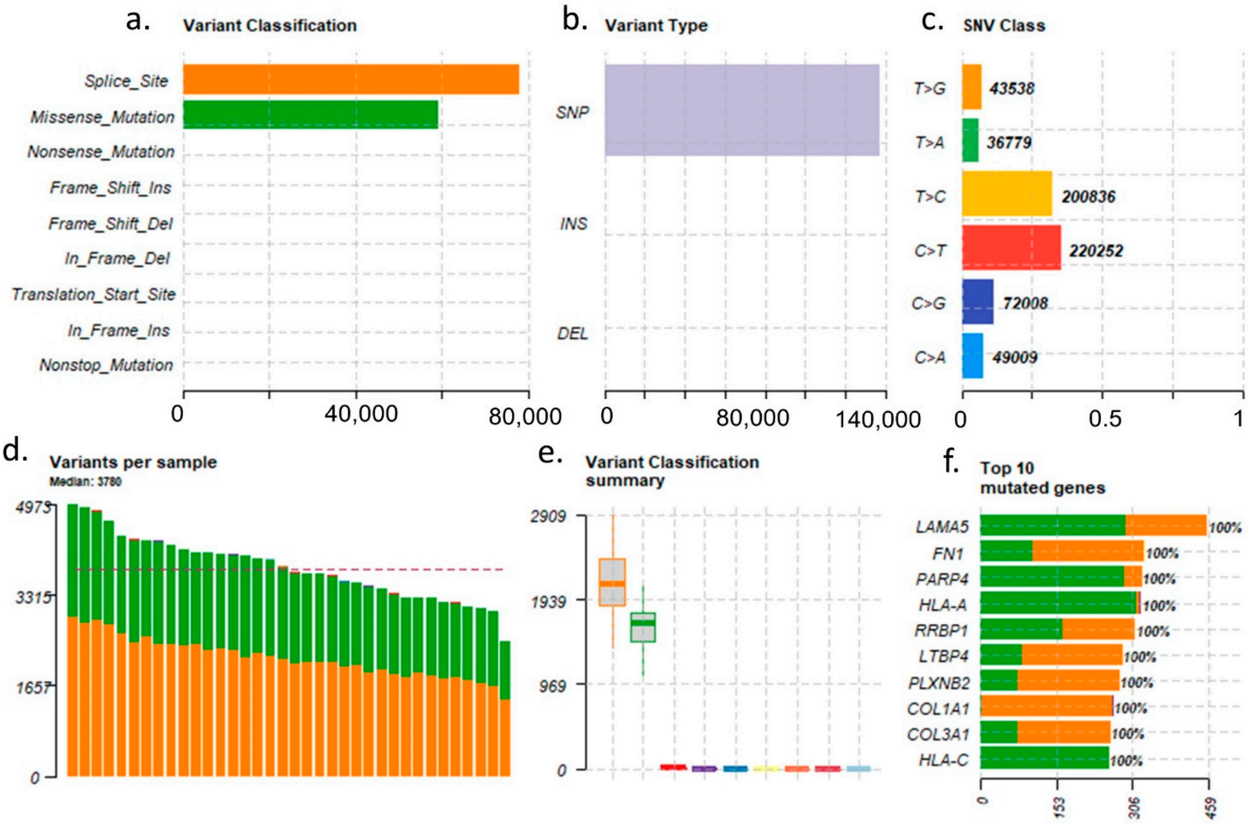

Supplementary Figure S5. Summary of Mutation Annotation Format (MAF) MAF files from normal endometrium samples. MAF files are used to store detected somatic variants. Representation of all single nucleotide variation (SNV) in the HGSC database:

s. SNV by classification: Frame-Shift deletion/insertion, In-Frame deletion/insertion, missense, non-sense or non-stop mutations, and splice site (x axis: total number);
t. SNV type: SNP, deletion or insertion (x axis: total number);
u. SNV class depending of nucleotide substitution (x axis: percentage);
v. SNVs per sample (y axis: total number);
w. Summary of SNV classification (based on a.), (y axis: total number);
x. Top 10 genes with number of SNVs (x axis: total number);

A.

Symbol	Cancer	Tube	OR	adjusted p-value
ANKRD36	62	0	Inf	1.1E-24
BRWD3	62	0	Inf	1.1E-24
CEP350	62	0	Inf	1.1E-24
CGNL1	62	0	Inf	1.1E-24
LRRC37A	62	0	Inf	1.1E-24
LRRC37A2	62	0	Inf	1.1E-24
LTBP1	62	0	Inf	1.1E-24
METTL2B	62	0	Inf	1.1E-24
MTMR6	62	0	Inf	1.1E-24
NBPF26	62	0	Inf	1.1E-24
POTEE	62	0	Inf	1.1E-24
STON2	62	0	Inf	1.1E-24
TTC21B	62	0	Inf	1.1E-24
WASHC2A	62	0	Inf	1.1E-24
ZNF600	62	0	Inf	1.1E-24
AC240274.1	61	0	Inf	1.4E-23
BOLA2-SMG1P6	61	0	Inf	1.4E-23
BZW1	61	0	Inf	1.4E-23
CDK11A	61	0	Inf	1.4E-23
DDX60L	61	0	Inf	1.4E-23
DSTYK	61	0	Inf	1.4E-23
FAM91A1	61	0	Inf	1.4E-23
GPD2	61	0	Inf	1.4E-23
IGHG1	61	0	Inf	1.4E-23
IGHG2	61	0	Inf	1.4E-23
METTL25	61	0	Inf	1.4E-23
NBEAL1	61	0	Inf	1.4E-23
NBPF1	61	0	Inf	1.4E-23
NOMO1	61	0	Inf	1.4E-23
PPCDC	61	0	Inf	1.4E-23
RPL36A	61	0	Inf	1.4E-23
RPL9	61	0	Inf	1.4E-23
SH3BGRL3	1	36	0	1.4E-23
SRP19	1	36	0	1.4E-23
TBC1D32	61	0	Inf	1.4E-23
TMED10	1	36	0	1.4E-23
ULK4	61	0	Inf	1.4E-23
WASH6P	61	0	Inf	1.4E-23
ZNF33B	61	0	Inf	1.4E-23
ZNF654	61	0	Inf	1.4E-23
ZNF91	61	0	Inf	1.4E-23

B.

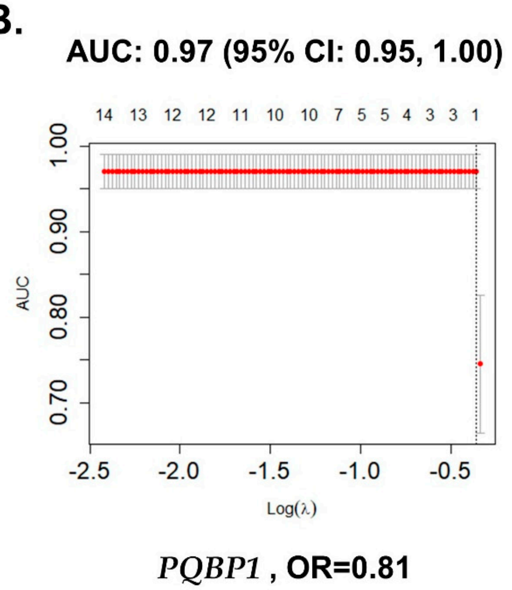

Supplementary Figure S6. Difference in SNV counts between EEC samples and normal endometrial samples.
A. In the univariate analysis 2,925 out of 15,792 unique genes had significant differences in SNV number between HGSC samples and tube samples (FDR adjusted p-value<0.001 to account for multiple comparisons). The table shows the 40 top genes.
B. The lasso multivariate regression model included all SNVs significant in the univariate analysis (N=2,925). Graphic representation of the multivariate lasso analysis: superior margin reflects number of variables; left margin reflects performance of the model measured in AUC (area under the curve); lower margin reflects lambda tunning parameter chose by cross-validation to optimize the model. The optimized AUC was 0.97 (95% CI: 0.95, 1.00), between the dotted lines. In the model, only differences in SNV number in *PQBP1* remained informative for prediction EEC, protecting against the presence of cancer (OR=0.81). Graphics were generated with R package glmnet.

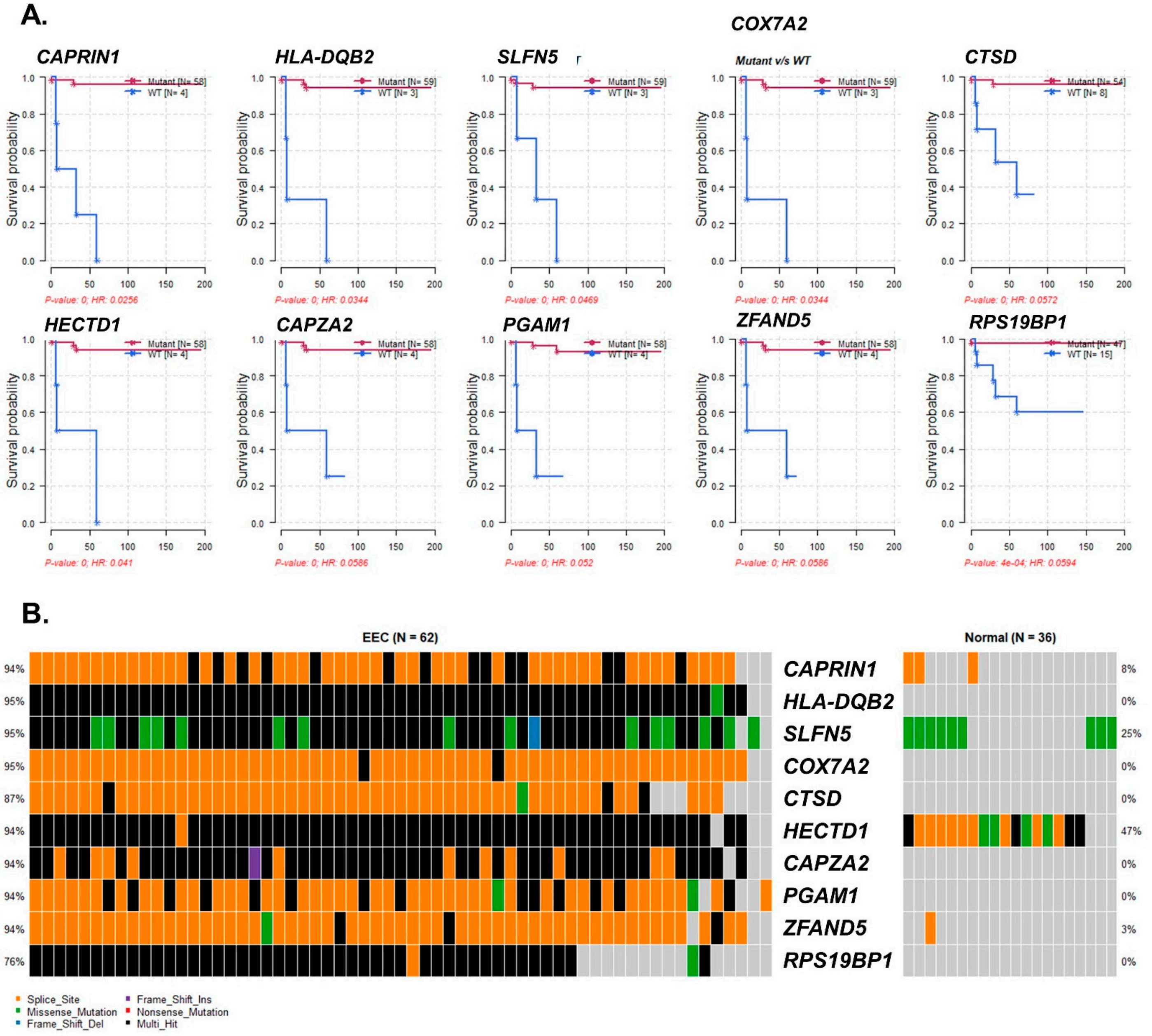

Supplementary Figure S7. Survival analysis of genes that had significant SNV differences between EEC and normal samples. A. Survival curves with respective hazard ratios (HR) of survival between the 10 top genes with differences between survival of wild type genes (WT) and genes with variations (Mut). Genes: CAPRIN1, HLA-DQB2, SLFN5, COX7A2, CTSD, HECTD1, CAPZA2, PGAM1, ZFAND5, RPS19BP1. **B.** Variant classification and number of SNV of these significant gene with different survivals. SNV classification: Frame-Shift deletion/insertion, In-Frame deletion/insertion, missense, non-sense or non-stop mutations, splice site, translation start site, and multi-hit.

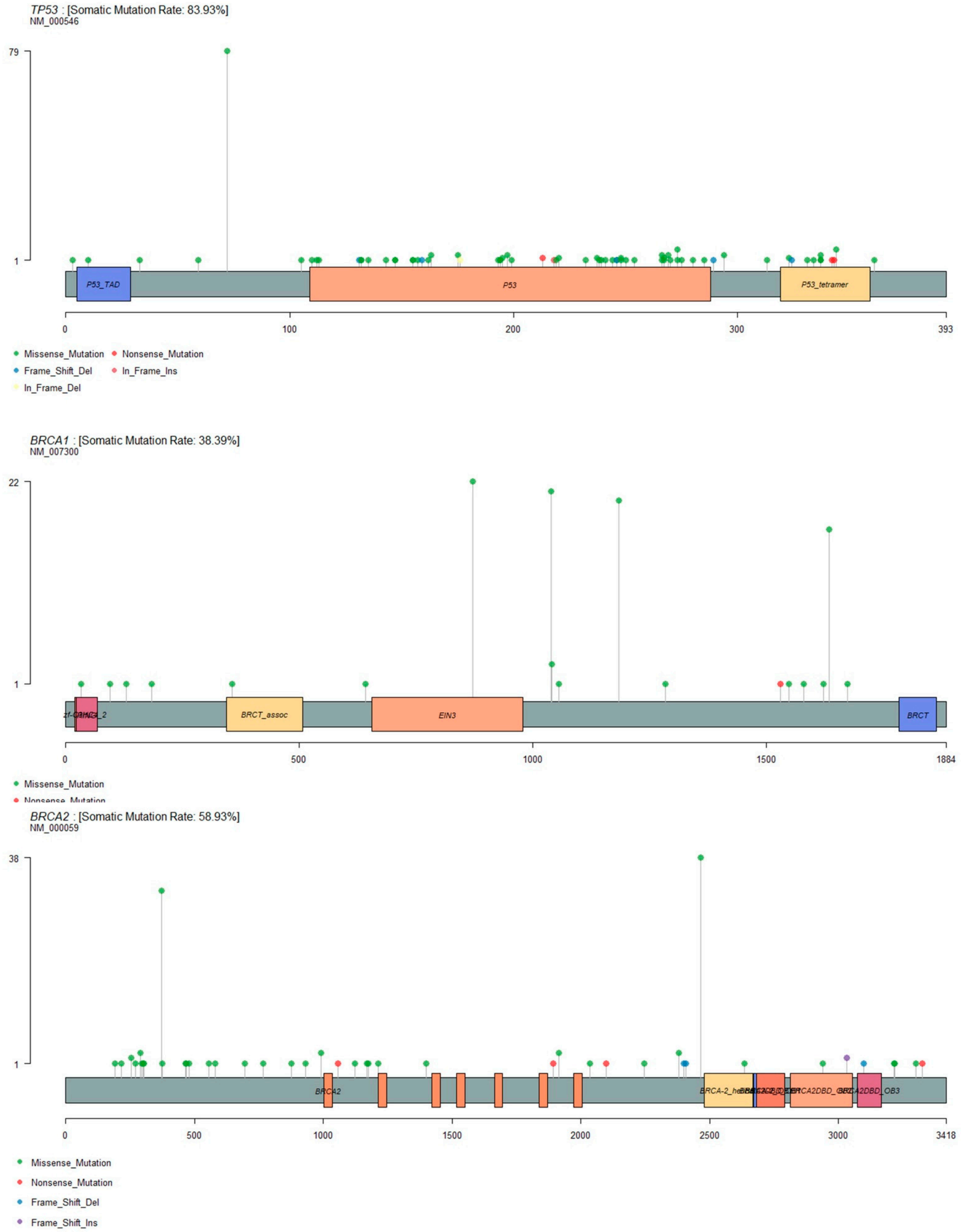
TP53 : [Somatic Mutation Rate: 83.93%]
NM_000546
79
1
P53_TAD
P53
P53_tetramer
0
100
200
300
393
Missense_Mutation
Nonsense_Mutation
Frame_Shift_Del
In_Frame_Ins
In_Frame_Del
BRCA1 : [Somatic Mutation Rate: 38.39%]
NM_007300
22
1
BRCT_assoc
EIN3
BRCT
0
500
1000
1500
1884
Missense_Mutation
BRCA2 : [Somatic Mutation Rate: 58.93%]
NM_000059
38
1
BRCA2
0
500
1000
1500
2000
2500
3000
3418
Missense_Mutation
Nonsense_Mutation
Frame_Shift_Del
Frame_Shift_Ins

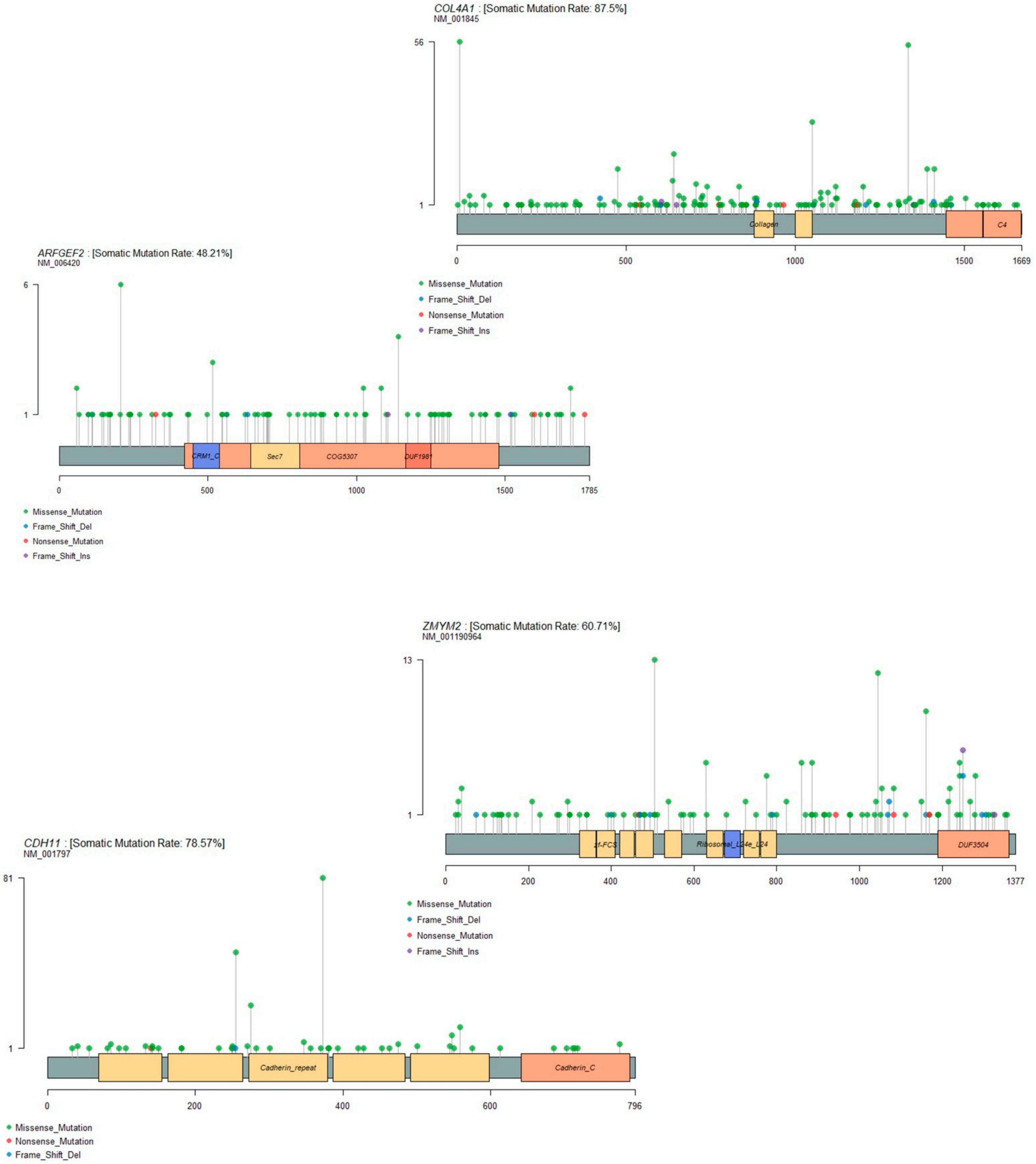

Supplementary Figure S8. Lollipop plots of the locations of variations of the genes that had significant SNV count and survival differences between HGSC and normal tube samples.

The lollipops represent variant locations and classification in genes: *COL4A1, ARFGEF2, ZMYM2*, and *PDPN*. Also added: *TP53, BRCA1* & *BRCA2*.

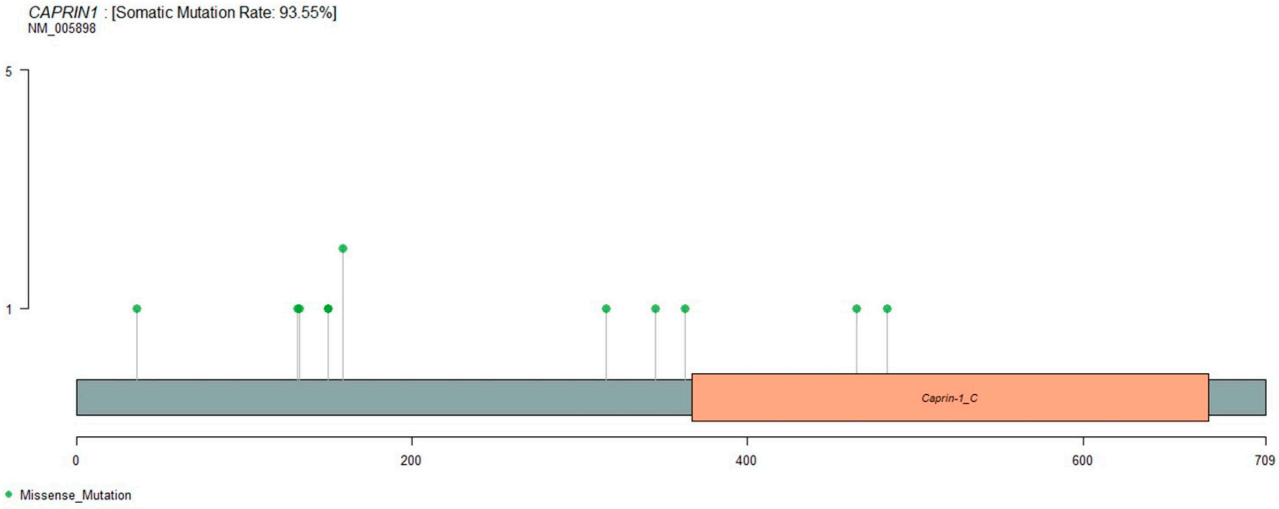
CAPRIN1 : [Somatic Mutation Rate: 93.55%]
NM_005898
5
1
Caprin-1_C
0
200
400
600
709
Missense_Mutation

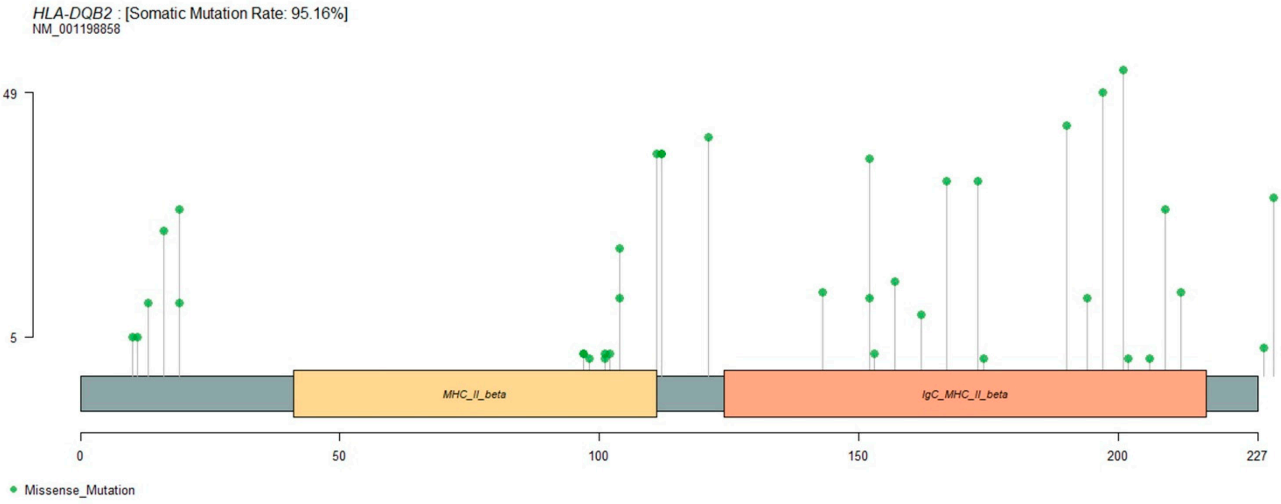
HLA-DQB2 : [Somatic Mutation Rate: 95.16%]
NM_001198858
49
5
MHC_II_beta
IgC_MHC_II_beta
0
50
100
150
200
227
Missense_Mutation

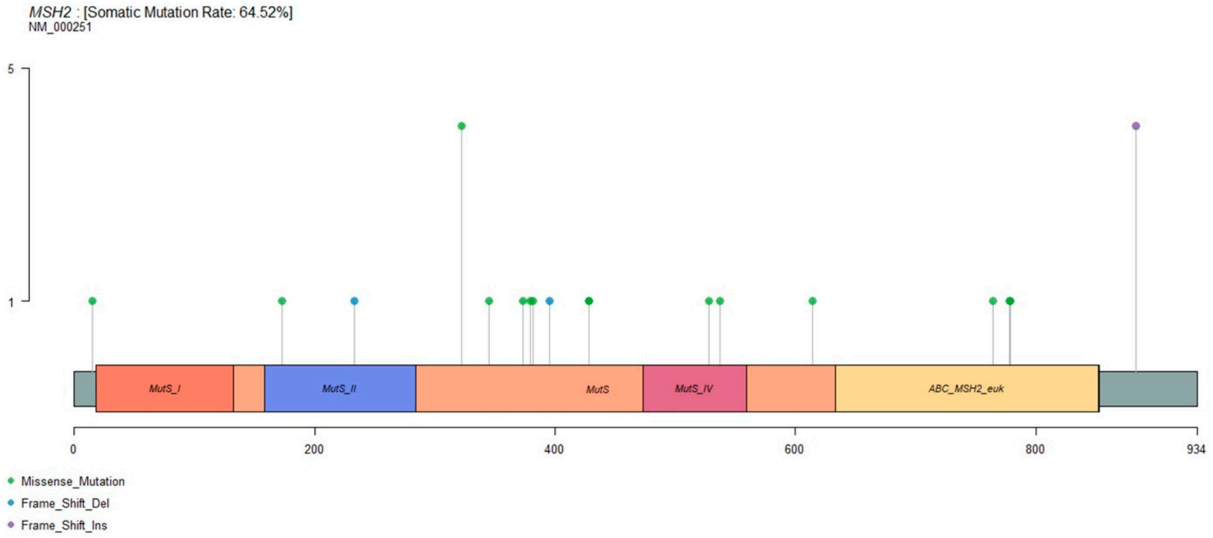
MSH2 : [Somatic Mutation Rate: 64.52%]
NM_000251
5
1
MutS_I
MutS_II
MutS
MutS_IV
ABC_MSH2_euk
0
200
400
600
800
934
Missense_Mutation
Frame_Shift_Del
Frame_Shift_Ins

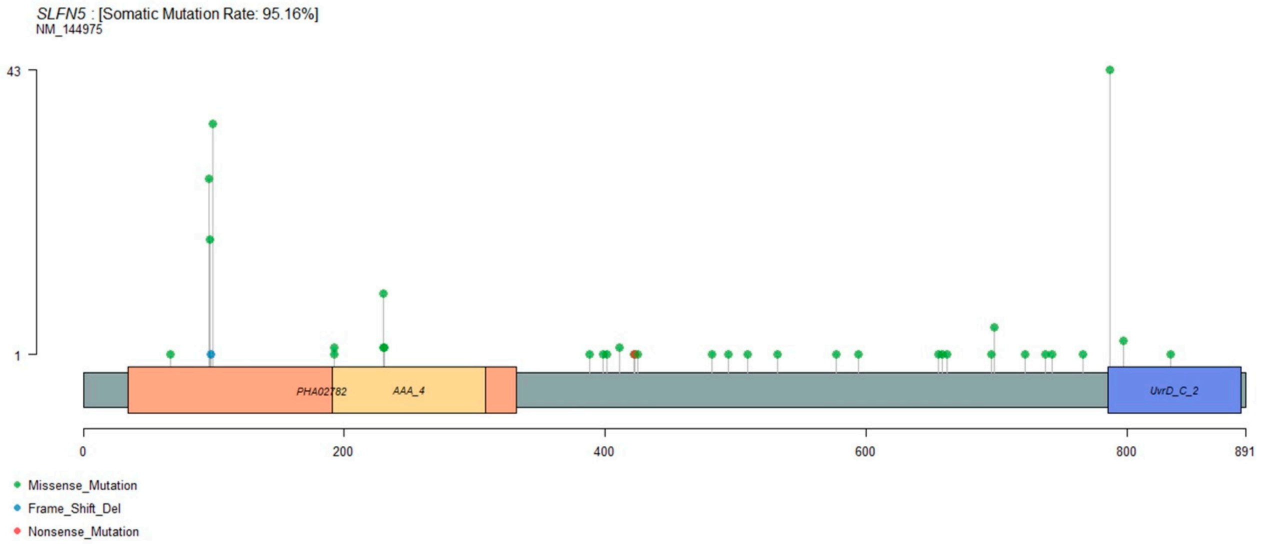
SLFN5 : [Somatic Mutation Rate: 95.16%]
NM_144975
43
1
PHA02782
AAA_4
UvrD_C_2
0
200
400
600
800
891
Missense_Mutation
Frame_Shift_Del
Nonsense_Mutation

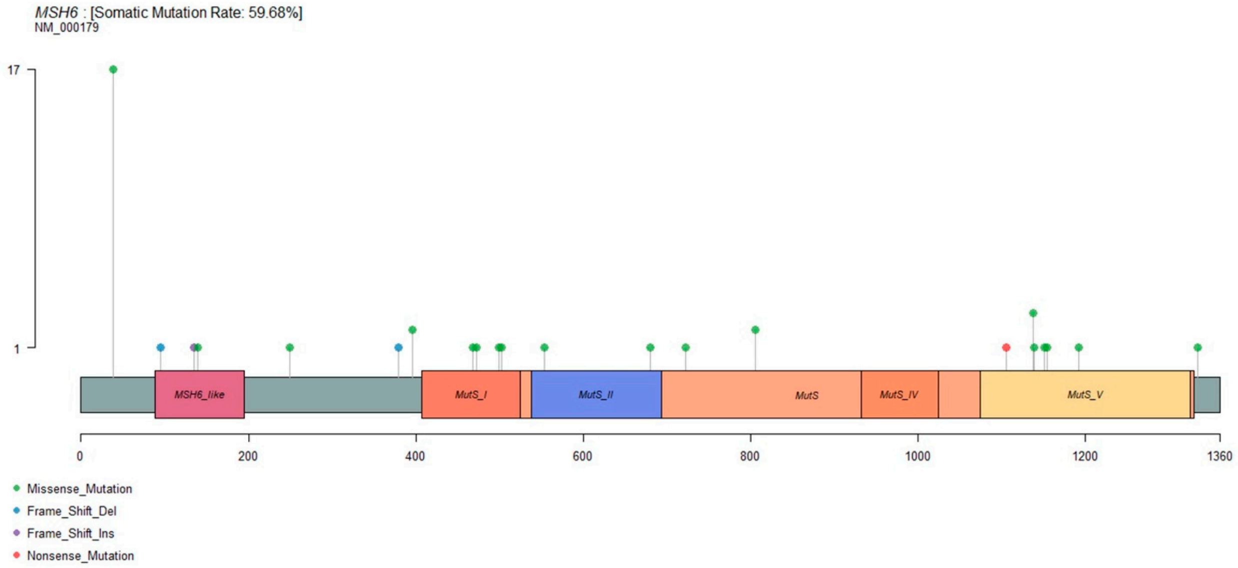
MSH6 : [Somatic Mutation Rate: 59.68%]
NM_000179
17
1
MSH6_like
MutS_I
MutS_II
MutS
MutS_IV
MutS_V
0
200
400
600
800
1000
1200
1360
Missense_Mutation
Frame_Shift_Del
Frame_Shift_Ins
Nonsense_Mutation

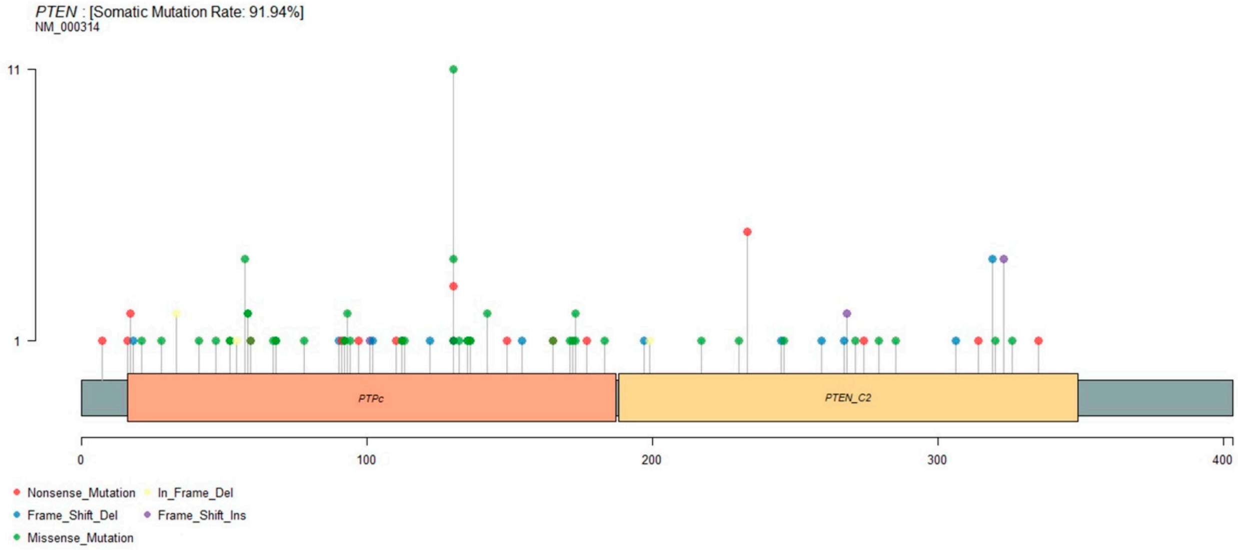
PTEN : [Somatic Mutation Rate: 91.94%]
NM_000314
11
1
PTPc
PTEN_C2
0
100
200
300
400
Nonsense_Mutation
In_Frame_Del
Frame_Shift_Del
Frame_Shift_Ins
Missense_Mutation

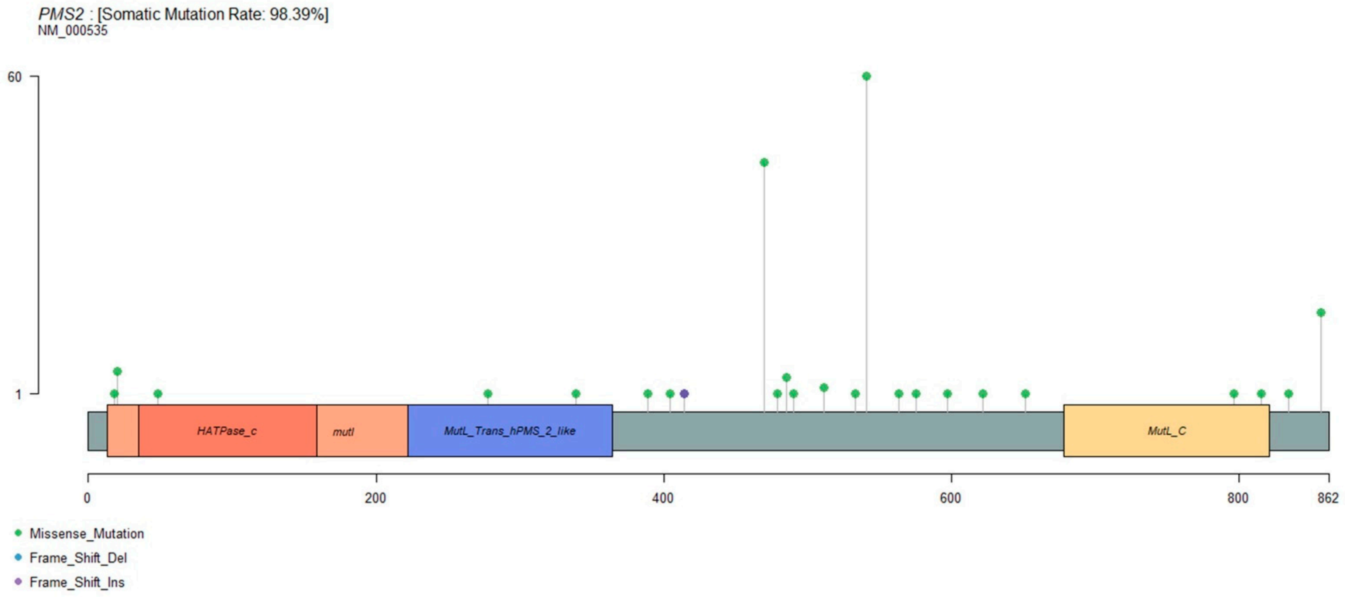

Supplementary Figure S9. Lollipop plots of the locations of variations of genes that had significant SNV count and survival differences between EEC and normal endometrial samples.

The lollipops represent variant locations and classification in genes: *CAPRIN1*, *HLA-DQB2*, and *SLFN5*. Then, we also added important genes in EEC, like *PTEN* (adjusted p<0.001), *MSH2* (adjusted p<0.001), *MSH6* (adjusted p=0.096) and *PMS6* (N.S.).

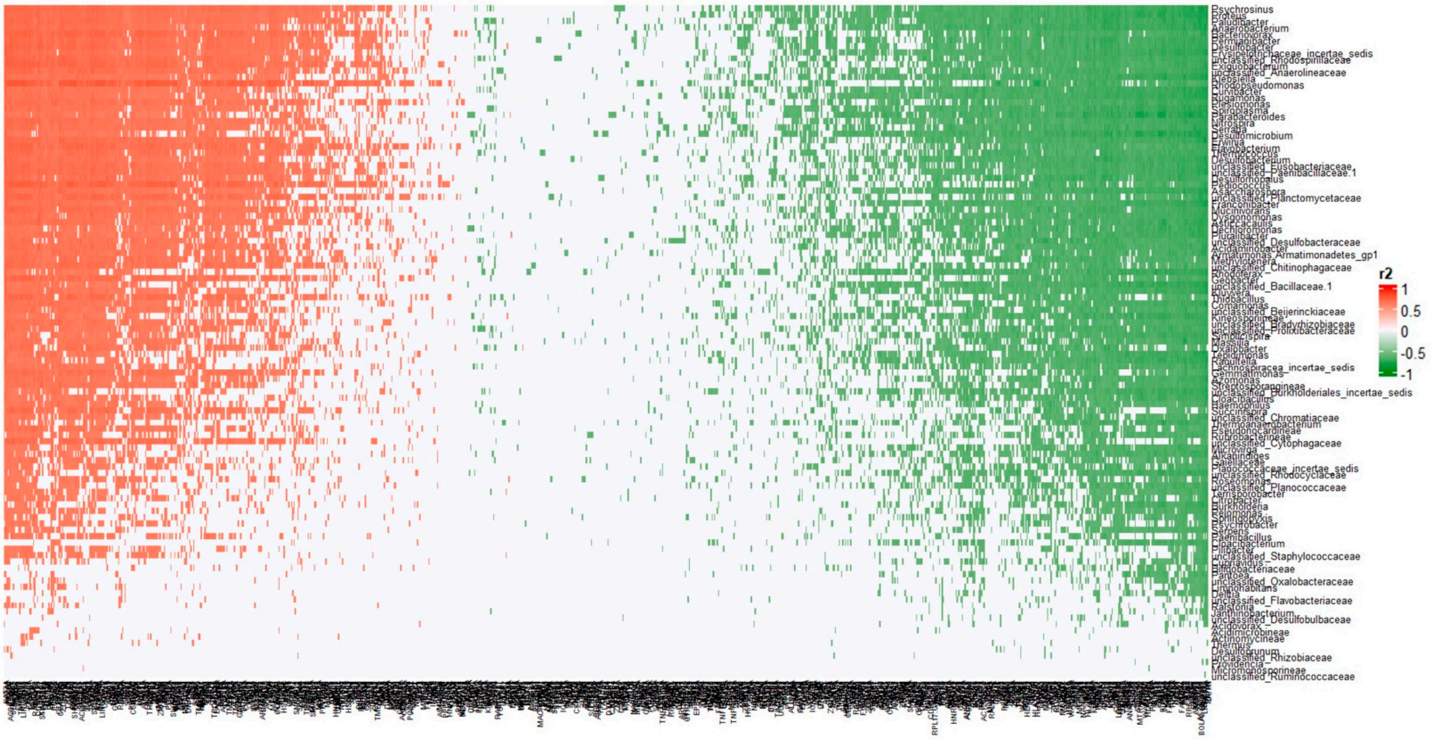

Supplementary Figure S10. Correlations of all ECC 16S rRNA counts with all EEC SNV counts.
The graphic represents all significant correlations between significantly different 16S rRNA gene expression counts between EEC and normal samples (N=112, FDR adjusted p-value<0.05), and significantly different gene-harboring SNV between the same samples (N=2,925, FDR adjusted p-value<0.001). Significant correlations were considered those with FDR adjusted p-value of <10^{-5}. There were 107 bacteria and 948 genes with significant correlations. Red represents significant direct correlations: more 16S rRNA counts, more gene SNVs. Green represents significant inverse correlations: more 16S rRNA counts, less SNVs.

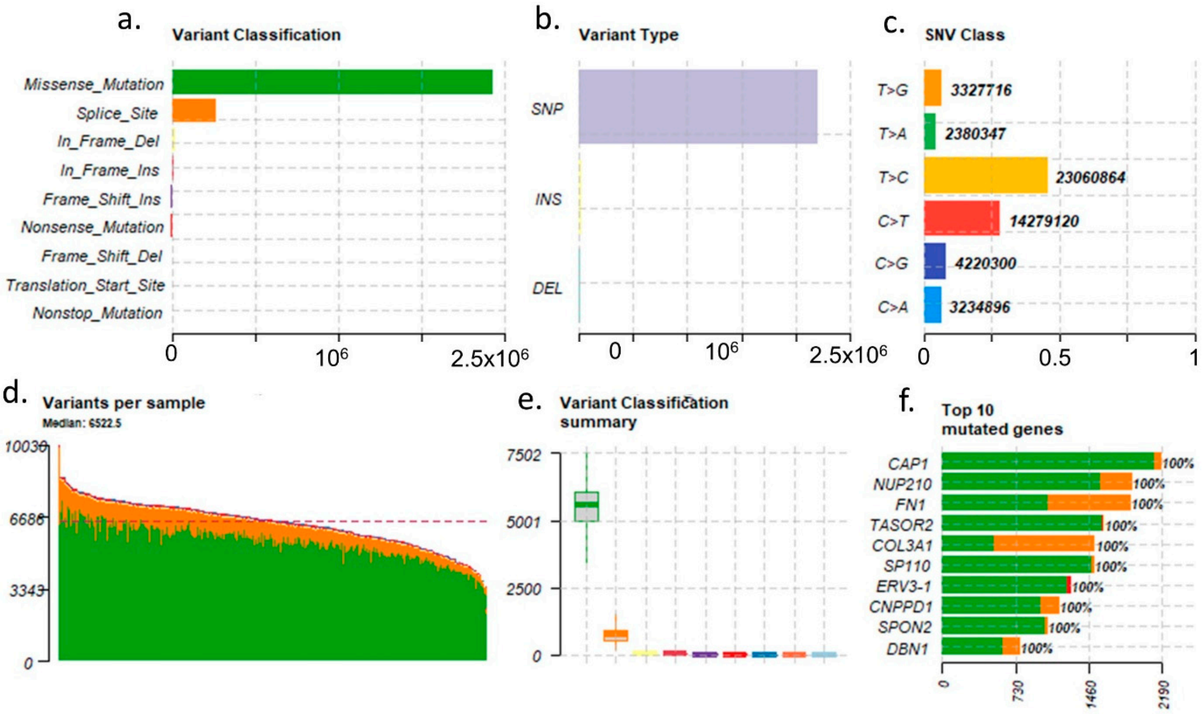

Supplementary Figure S11. Summary of Mutation Annotation Format (MAF) MAF files from TCGA HGSC samples. MAF files are used to store detected somatic variants. Representation of all single nucleotide variation (SNV) in the HGSC database:

y. SNV by classification: Frame-Shift deletion/insertion, In-Frame deletion/insertion, missense, non-sense or non-stop mutations, and splice site (x axis: total number);
z. SNV type: SNP, deletion or insertion (x axis: total number);
aa. SNV class depending of nucleotide substitution (x axis: percentage);
bb. SNVs per sample (y axis: total number);
cc. Summary of SNV classification (based on a.), (y axis: total number);
dd. Top 10 genes with number of SNVs (x axis: total number);

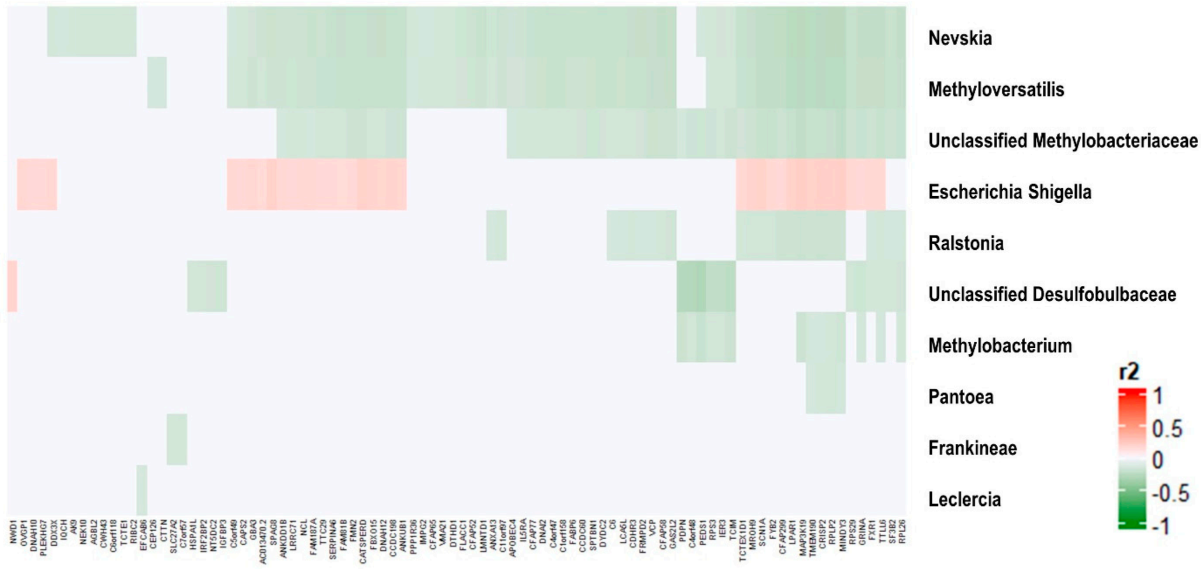

Supplementary Figure S12. Validation of correlations between 16s rRNA counts and SNV counts with TCGA HGSC database. The graphic represents the correlation (measured in r^2) between significantly different 16S rRNA gene expression counts between HGSC and tubal samples in the UI dataset and determined in TCGA (N=13), and significantly SNV counts between the same samples in UI determined in TCGA: 592 out of 593. In the initial UI dataset, significant correlations were observed between 11 different 16S rRNA genes and 160 different genes harboring SNVs. In TCGA HGSC, significant correlations were observed between 10 (out of the 11) 16S rRNA genes and 90 (out of the 123) genes harboring SNVs. Red represents significant direct correlations: more 16S rRNA counts, more SNV gene counts. Green represents significant inverse correlations: more 16S rRNA counts, less SNV counts.

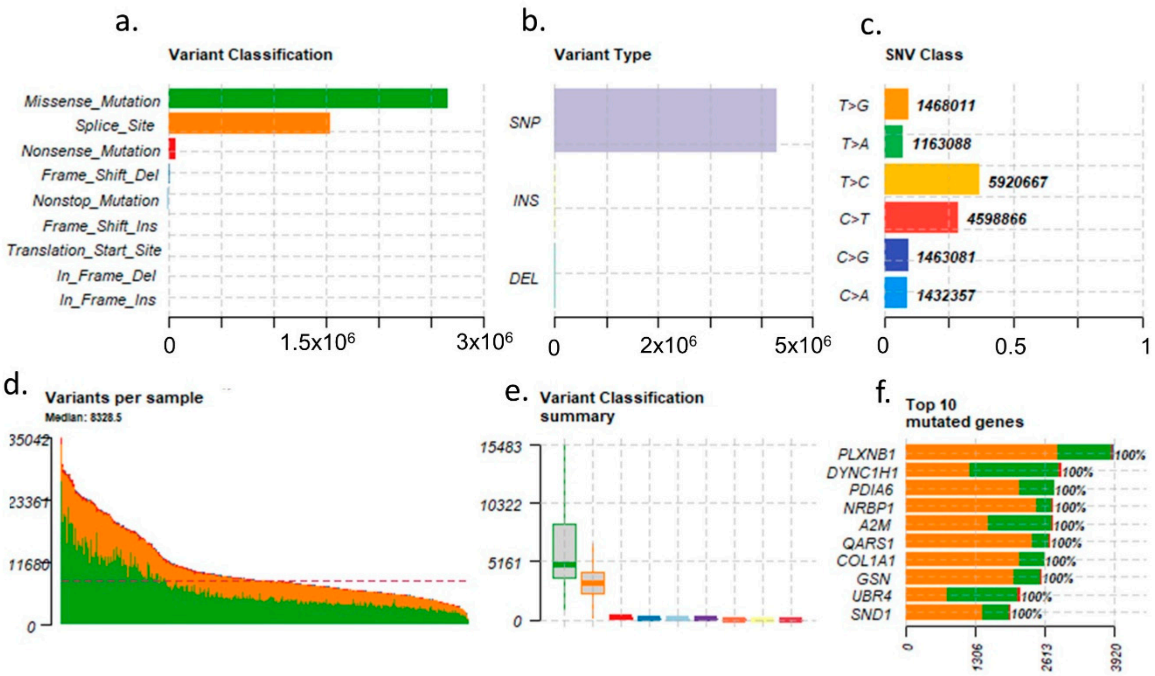

Supplementary Figure S13. Summary of Mutation Annotation Format (MAF) MAF files from TCGA EEC samples. MAF files are used to store detected somatic variants. Representation of all single nucleotide variation (SNV) in the HGSC database:

ee. SNV by classification: Frame-Shift deletion/insertion, In-Frame deletion/insertion, missense, non-sense or non-stop mutations, and splice site (x axis: total number);
ff. SNV type: SNP, deletion or insertion (x axis: total number);
gg. SNV class depending of nucleotide substitution (x axis: percentage);
hh. SNVs per sample (y axis: total number);
ii. Summary of SNV classification (based on a.), (y axis: total number);
jj. Top 10 genes with number of SNVs (x axis: total number);

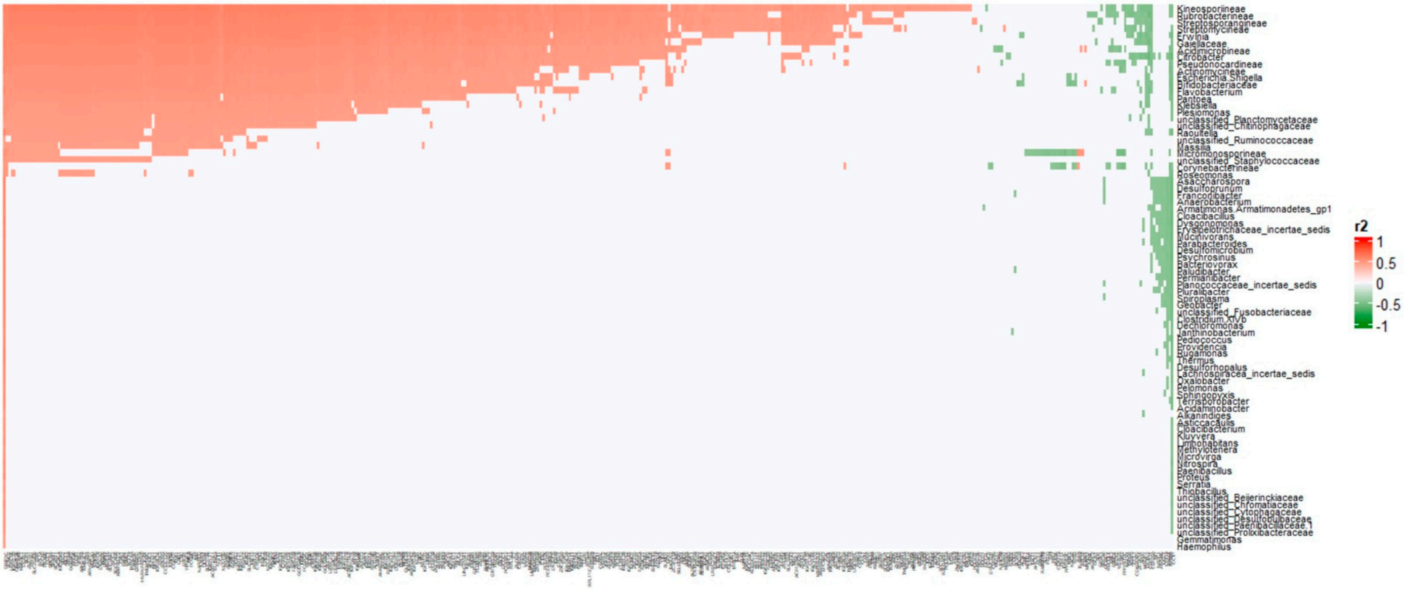

Supplementary Figure S14. Validation of correlations between 16s rRNA counts and SNV counts with TCGA EEC database. The graphic represents the correlation (measured in r^2) between significantly different 16S rRNA gene expression counts between EEC and tubal samples in the UI dataset and determined in TCGA (N=107 out of 112), and significantly SNV counts between the same samples in UI determined in TCGA: 2831 out of 2925. In the UI dataset, significant correlations were observed between 107 bacteria and 948 genes with SNVs (FDR adjusted p-value<10^{-5}). In TCGA, significant correlations were observed between 79 different 16S rRNA genes and 447 different genes harboring SNVs (p-value<10^{-5}). We represented all significant correlations. Red represents significant direct correlations: more 16S rRNA counts, more SNV gene counts. Green represents significant inverse correlations: more 16S rRNA counts, less SNV counts.

WP4803 Ciliopathies (a)

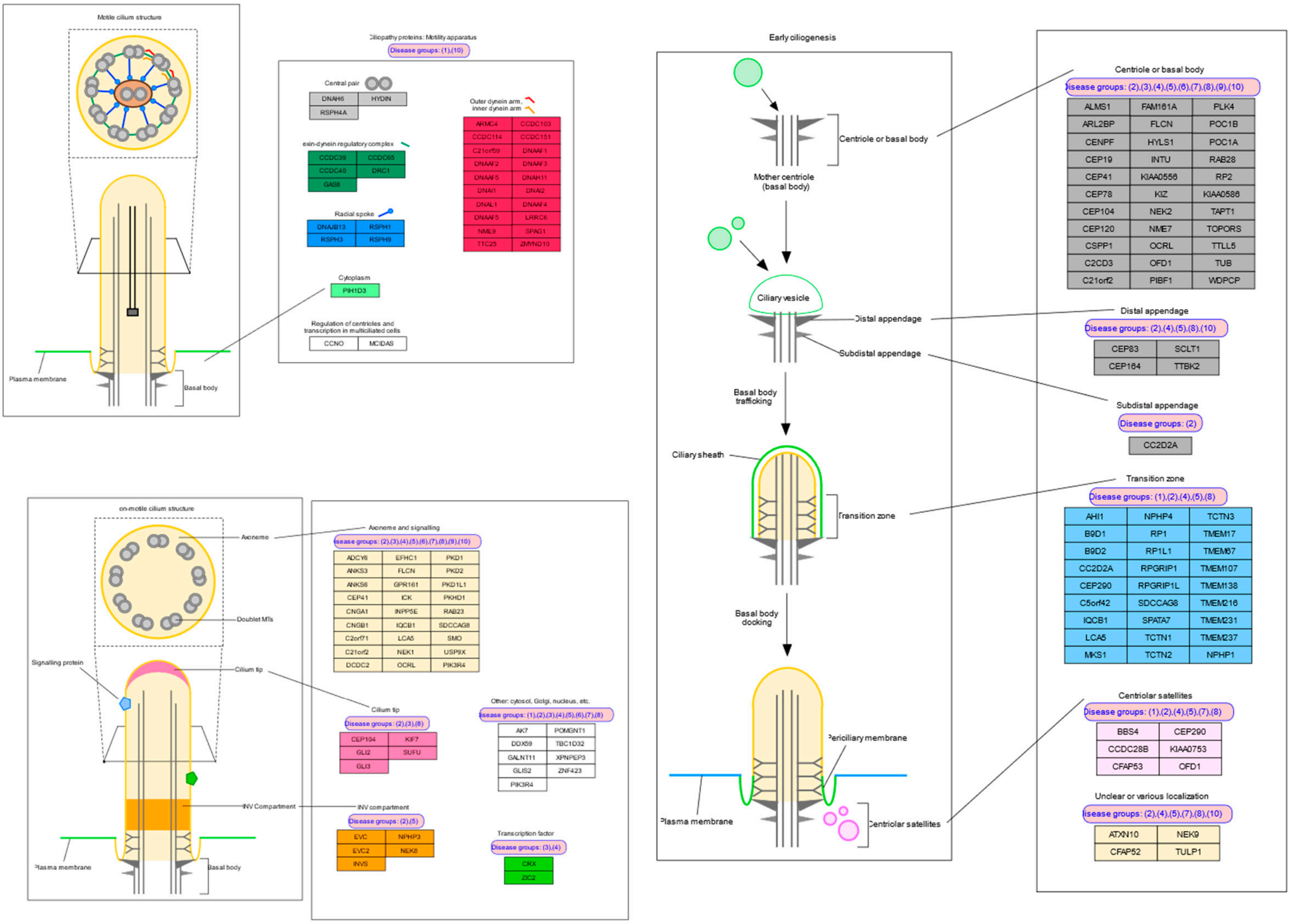

WP4803 Ciliopathies (b)

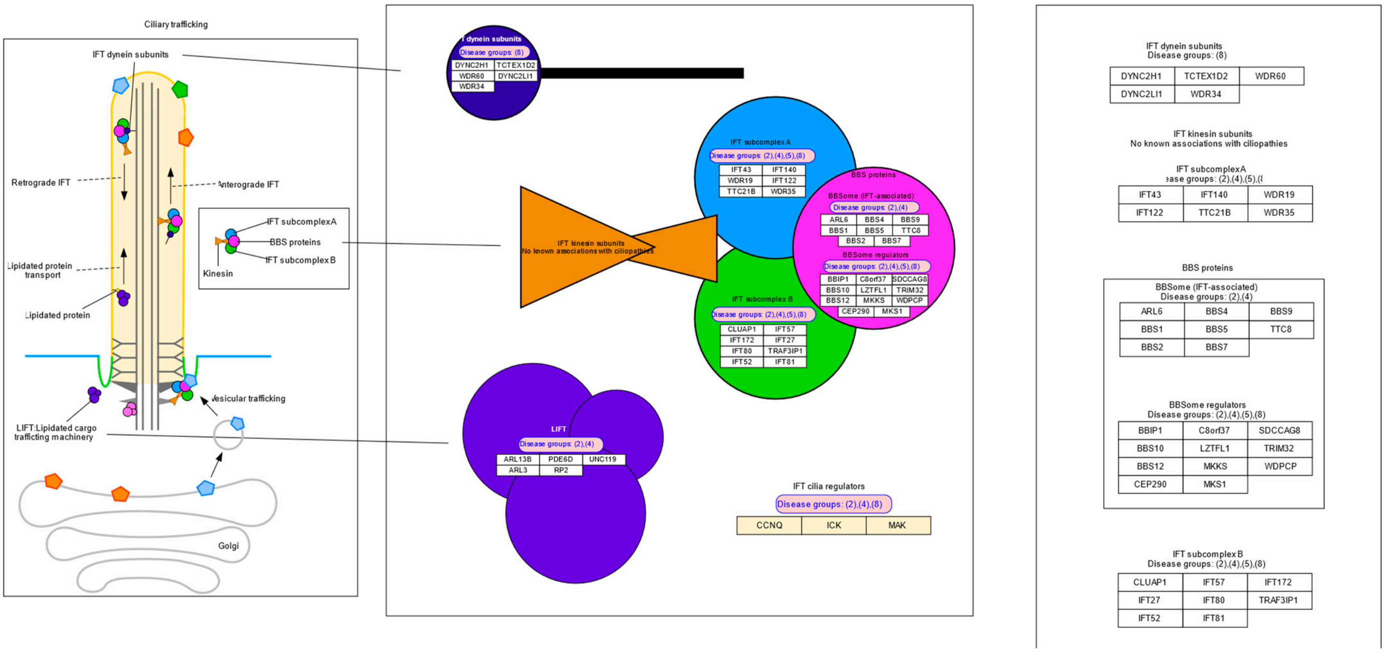

WP4538 Regulatory circuits of the STAT3 signaling pathway (c)

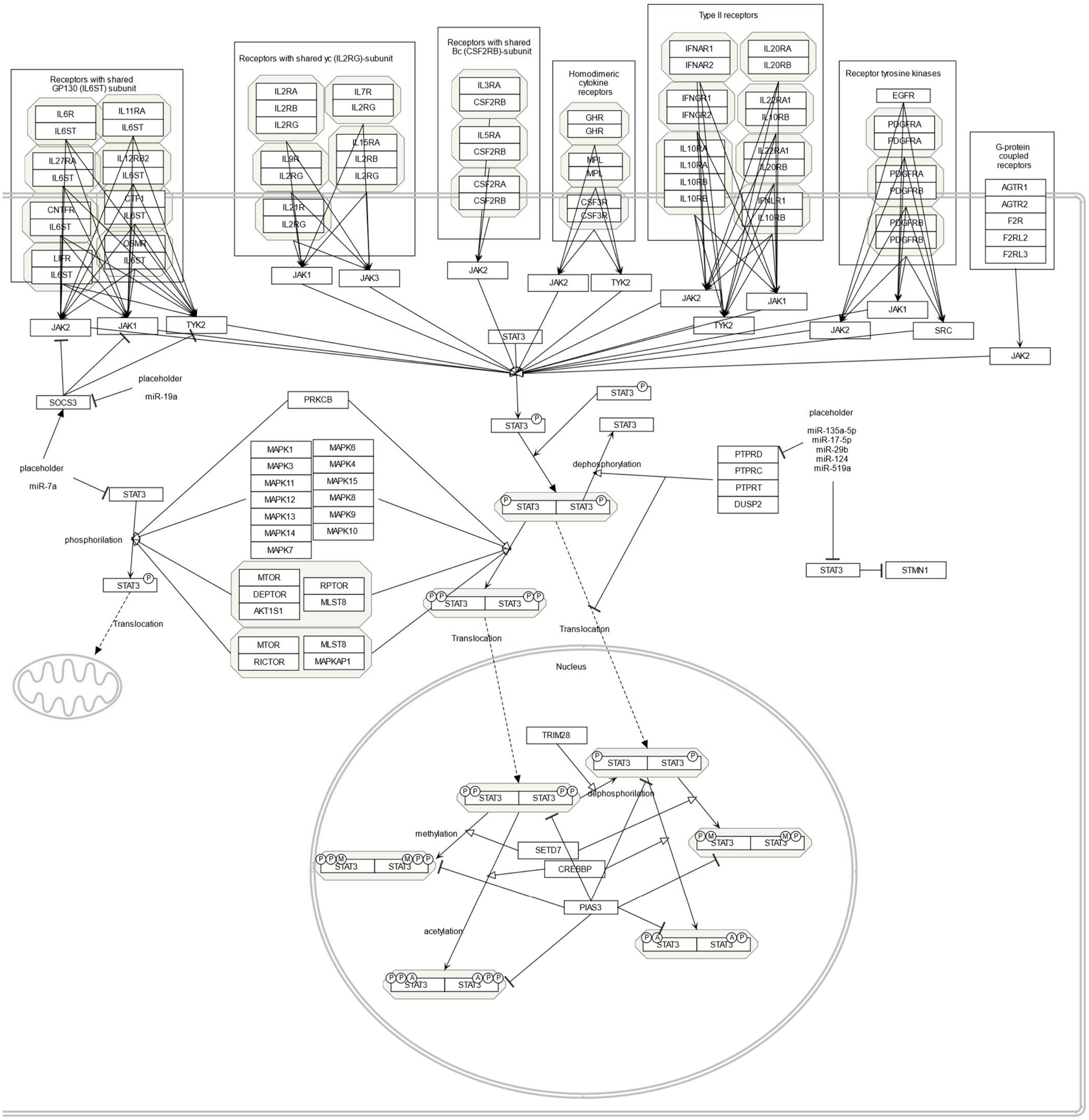

WP3888 VEGFA-VEGFR2 signaling (d)

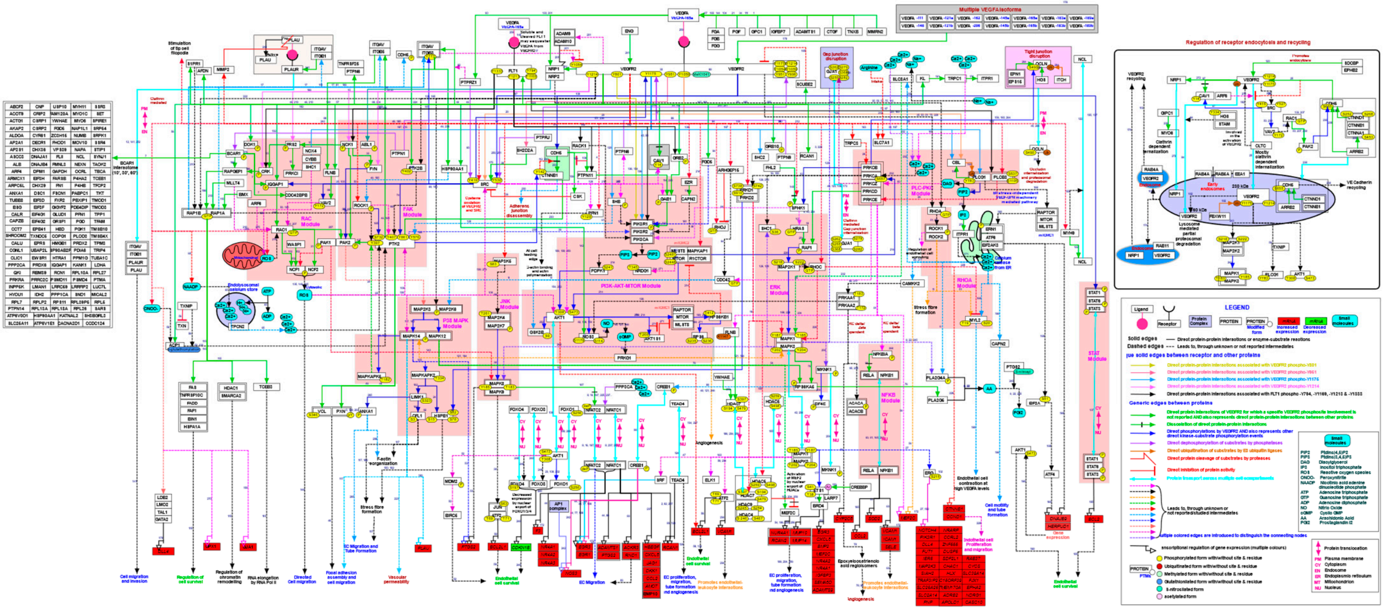

WP2272 Pathogenic Escherichia coli infection (e)

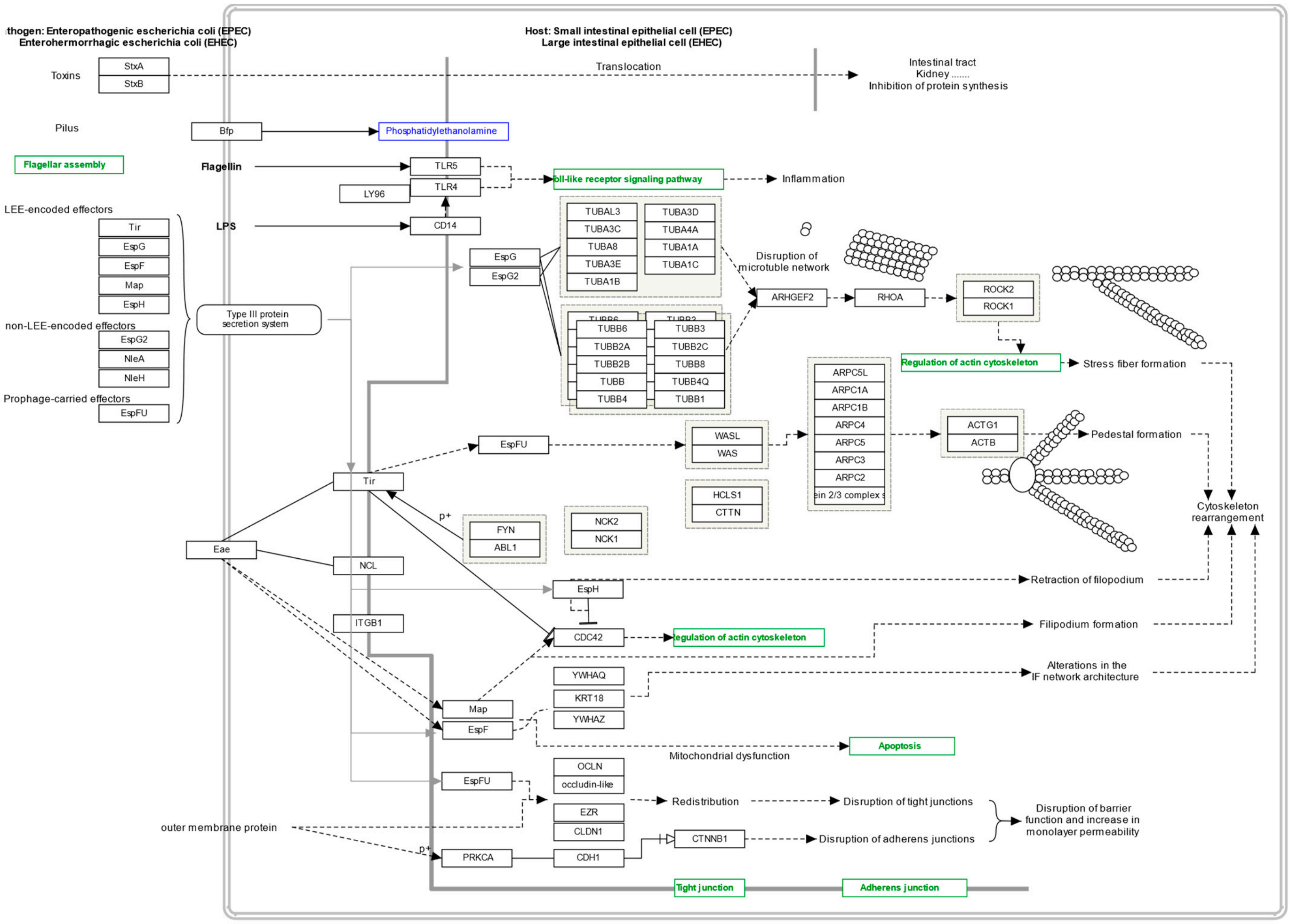

WP4853 Linoleic acid metabolism affected by SARS-CoV-2 (f)

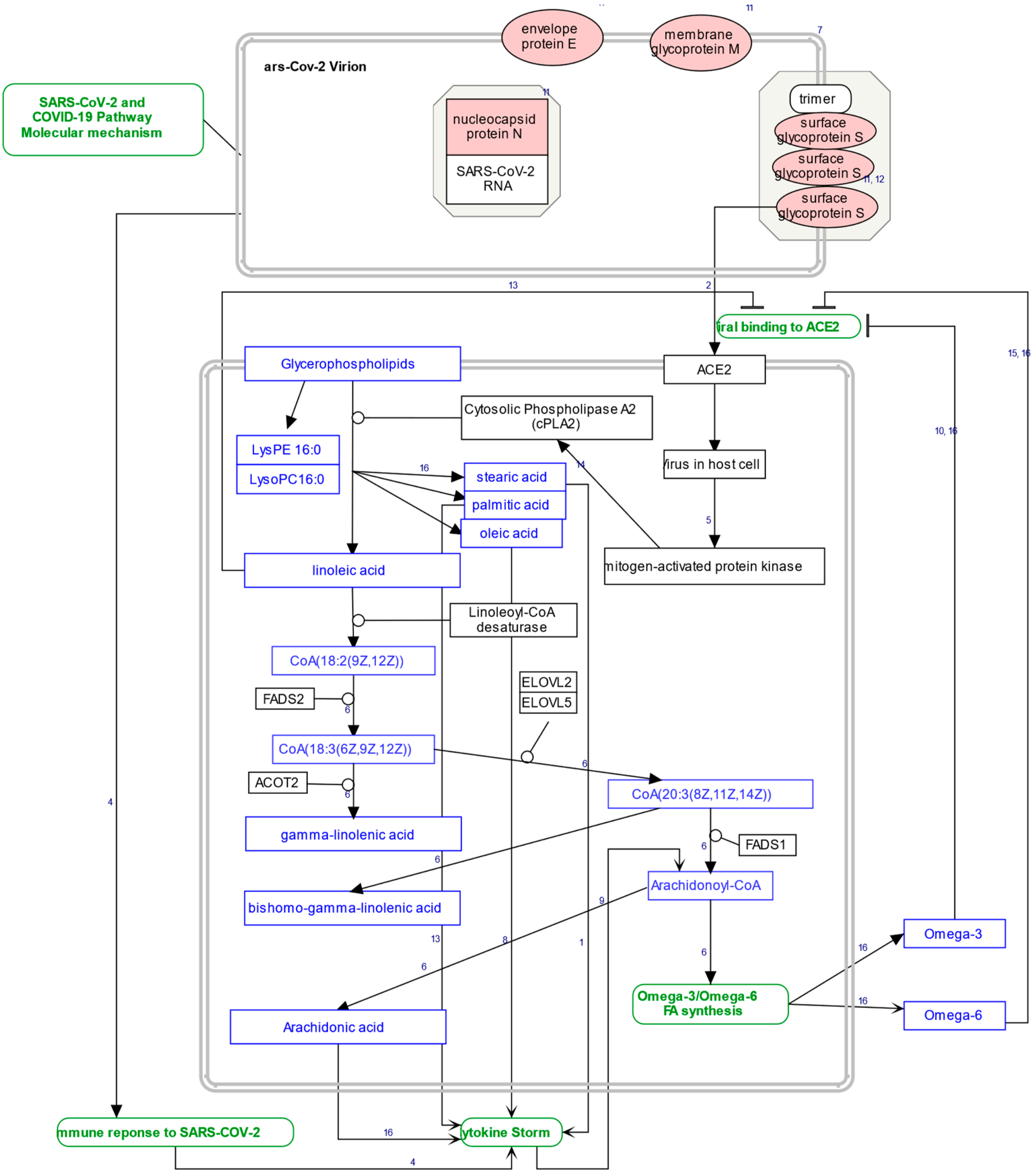

Supplementary Figure S15. Enrichment pathway analysis of genes of genes with SNVs that were significantly correlated to 16S rRNA transcripts in HGSC and EEC. Significant enriched WikiPathways for the genes with differential SNVs in the HGSC database:

WP4803 Ciliopathies (a,b)

WP4538 Regulatory circuits of the STAT3 signaling pathway (c)

WP3888 VEGFA-VEGFR2 signaling (d)

WP2272 Pathogenic Escherichia coli infection (e)

WP4853 Linoleic acid metabolism affected by SARS-CoV-2 (f)
